# Liver Antioxidants in Relation to Beak Morphology, Gizzard Size and Diet in the Common Eider *Somateria mollissima*

**DOI:** 10.3390/antiox8020031

**Published:** 2019-01-31

**Authors:** Anders Pape Møller, Karsten Laursen, Filiz Karadas

**Affiliations:** 1 Ministry of Education Key Laboratory for Biodiversity Science and Ecological Engineering, College of Life Sciences, Beijing Normal University, Beijing 100875, China; 2 Ecologie Systématique Evolution, Université Paris-Sud, CNRS, Agro ParisTech, Université Paris-Saclay, F-91405 Orsay CEDEX, France; 3 Department of Bioscience, Aarhus University, Grenåvej 14, Kalø, DK-8410 Rønde, Denmark; kl@bios.au.dk; 4 Department of Animal Science, Faculty of Agriculture, Van Yuzuncu Yil University, 65080 Van, Turkey; filizkaradas07@gmail.com

**Keywords:** antioxidants, α-tocopherol, retinol, carotenoids, coenzyme Q_10_, eiders

## Abstract

Antioxidants in the liver are particularly abundant in capital breeders that rely on stored resources for egg production. Capital breeders like eider (hereafter common eider) *Somateria mollissima* have disproportionately large livers with low levels of coenzyme Q_10_ when compared to other bird species. Concentrations of total carotenoids and vitamin E in the livers of eiders were smaller than predicted for similarly sized bird species. Eiders with high body condition estimated as body mass relative to skeletal body size had high levels of total carotenoids and low levels of coenzyme Q_10_. The concentration of total carotenoids per gram of liver increased with age, and vitamin E and total carotenoids accumulated during the winter onwards from February to peak at the start of incubation in April. Total vitamin E, total carotenoids, and coenzyme Q_10_ per gram of liver decreased with increasing beak volume. The size of the empty gizzard increased with increasing liver mass but decreased with total carotenoids and coenzyme Q_10_. The main components of the diet were blue mussels *Mytilus edulis* (40%), draft whelk *Nassarius reticulatus* (27%), and periwinkle *Littorina littorea* (10%). The concentration of vitamin E increased with the number of razor clams *Ensis* sp. and draft whelks in the gizzard and the concentration of total carotenoids increased with the number of beach crabs *Carcinus maenas*. These observations are consistent with the hypothesis that eiders are limited in their levels of antioxidants through food limitation. Furthermore, they imply that diet and morphological characters involved in food acquisition and processing are important determinants of the level of antioxidants in the liver.

## 1. Introduction

Antioxidants play an important role in wild animals with vitamin A and E, carotenoids, and coenzyme Q_10_ controlling reactive oxygen species [1,2]. Diet and food choice may influence antioxidant concentration, but in most bird species it is unclear whether they ultimately affect survival and reproduction. Hence, more research is needed. Diet and food choice will influence the concentration of antioxidants, and if they occur in limited supply, they may ultimately affect survival and reproduction. Nutritional requirements of birds change during the annual cycle, being the highest during the reproductive cycle just before laying because all resources required for adequate egg development are determined by pre-laying food acquisition [2,3]. In capital breeders like the common eider *Somateria mollissima,* the female spends the entire incubation period relying on resources stored before and during laying and incubation [4,5]. Within any given stage of life, the environments in which the capital breeders are located have profound effects on nutrition [3], as reflected in both the quantity and quality of food consumed to achieve an optimal diet and for maintenance of their body condition. In the wild, foraging is often the predominant activity in which a bird engages. For example, many migrating birds spend in excess of 80% of daylight hours during winter in search of food [3]. 

The balance between tissue antioxidants and prooxidants is responsible for normal embryonic development, but also for post-hatch offspring viability [1,2]. Avian maternal nutrition is the major determinant of the health and development of offspring before and also after hatching [2,6]. Previous studies have shown a significant transfer of antioxidants such as selenium [7] and carotenoids [8,9,10] from the maternal diet to progeny tissue. Extensive transfer of antioxidants from yolk to avian embryonic tissue takes place during the last week of embryonic development in line with increasing lipid unsaturation, with maximum transfer occurring just after hatching [11]. Studies of birds have suggested that low levels of antioxidants in females result in fewer eggs laid [12]. Moreover, increasing antioxidant supplementation to mothers resulted in more antioxidant transfer to eggs, with beneficial consequences for certain offspring traits [13]. Finally, antioxidant concentrations are higher in yolk than in maternal tissue, suggesting that these compounds are actively allocated from the mother to specific egg tissues [6,14,15]. The incorporation of antioxidants from yolk into the developing tissue of embryo and chick (e.g., liver, blood) may contribute to the antioxidant defense of the offspring [8,16,17,18,19]. Artificial injection of antioxidants into the egg has been shown to enhance acquired immunity with no apparent adverse consequences for other offspring traits (the first such experimental study was reported by Saino et al. [20]). Studies of poultry and fish in captivity have shown that egg antioxidants improve egg hatchability, body mass, and osteometric growth rates, immunity, behavior, and viability in the early post-hatch period [14,21].

Moreover, Vitamin E levels in day-old chick livers have been directly related to chick viability [8]. Coenzyme Q_10_ is a key component in the inner mitochondrial membrane, where it plays an important role in oxidative phosphorylation [22,23]. It is also present in plasma lipoproteins, where it acts as an important antioxidant [24]. Serum or liver malonaldehyde (MDA), which is a product of lipid peroxidation, was significantly decreased by coenzyme Q_10_ supplementation [25]. Coenzyme Q_10_ in its reduced form acts as a free radical scavenger [26], and it is preferred over α-tocopherol [27]. Coenzyme Q_10_ is involved in the prevention of lipid peroxidation and in the regeneration of other endogenous antioxidants [28]. There are only a few studies on the effects of feeding long-lived animals with dietary coenzyme Q_10_ and its distribution in the tissue [29]. Hence there is a need for additional research on the content of coenzyme Q_10_ in the natural diet, but also in tissues of wild birds. 

Common eiders are capital breeders that build up their body reserves during winter for subsequent breeding [4]. When females arrive from the wintering grounds in Denmark to the breeding areas in the Northern Baltic during spring migration, they enter colonies and produce a clutch of 4–5 eggs that are incubated for about 26 days without the females leaving the nest to feed. Studies in the Wadden Sea show that wintering numbers of eiders are positively related to mussel stocks, and individuals feeding on mussels have better body conditions than those taking other types of prey, which makes it attractive for eiders to feed on mussel beds [30,31,32]. Blue mussel *Mytilus edulis* can affect the feeding conditions of eiders by increasing flesh content (a standard term for the amount of soft tissue in a mussel that can be consumed by eiders) and by increasing the size of mussel stocks. 

The objectives of this study were (1) to quantify the interspecific allometric relationship between liver mass and body mass in order to assess the relative position of the common eider in terms of liver mass and to test whether common eiders have significantly larger livers than other species for a given body size, as expected from their strenuous diving habit; (2) to assess the relationship between liver size and concentrations of total antioxidants, vitamin E, vitamin A, and coenzyme Q_10_, respectively, and beak volume, which is the feature of the beak that affects the ability of individuals to capture larger prey, a larger amount and process a larger quantity of food. Thus, we predicted a positive relationship between the amounts of antioxidants and size of the beak. Objective (3) was to quantify the relationship between liver size and antioxidant concentrations, respectively, gizzard size and antioxidant concentration, and the relationship between liver size and antioxidant concentrations, respectively, and diet. We expect positive relationships if there is no trade-off between one category of antioxidants and another. 

## 2. Materials and Methods

### 2.1. Liver Mass and Body Mass in Different Species of Birds

A colleague Johannes Erritzoe obtained and dissected the liver from 178 species of birds during 1960–2018 in Jutland, Denmark. Following removal of the liver, it was weighed on a precision balance scale to the nearest 0.01 g, as was the body mass of the same specimens. 

### 2.2. Study Sites and Study Samples

We recorded morphological characters and liver samples for 162 eiders following collection by Danish hunters during winter and spring (10 February–11 May) 2016–2017 (2016: *N* = 56; 2017: *N* = 106). Dates for all measurements were in October (1 = 1 October) because molting is finished by the end of September. Eiders were weighed (with heavier individuals for a given body size being considered to be in better condition), sexed (81 males and 81 females), and aged. Livers were frozen immediately upon arrival at the lab, where they were subsequently analyzed. We included males and females because the values for females could then be compared with those for males that do not lay and incubate eggs. Females were aged up to the third calendar year, and males up to the sixth calendar year. All eiders were sexed and aged using standard plumage characteristics [33]. We recorded measurements of eiders in the laboratory and performed antioxidant analyses.

We recorded the number of blue mussels, cockles *Cerastoderma edule,* razor clams *Ensis* sp., other mussels, periwinkles *Littorina littorea,* draft whelks *Nassarius reticulatus*, conchs *Buccinius undatum*, beach crabs *Carcinus maenas,* and other items, in total nine categories from the 162 eiders in this study. Intact food items were identified to species or species groups and their maximum length measured (mm). For broken food items, we used a reference collection for identification and assessment of size (in 5 mm intervals). We estimated the mean and the maximum lengths for each prey group in each gizzard. 

We obtained permits from the Ministry of the Environment, Denmark, to perform the study (SN-302-009, SNS-3446-00103, NST-3446-00018, NST-3465-00007).

### 2.3. Sample Extraction and HPLC Analysis

Carotenoids, vitamins A and E, and coenzyme Q_10_ were extracted from liver samples as described before for fat-soluble antioxidants for the 162 eiders included in this study [34,35]. While total carotenoids may only have a weak antioxidant effect overall, we included a range of different antioxidants in order to search for the most important components. Briefly, about 200 mg liver sample was mixed with a 5% solution (w/v in H_2_O) of NaCl (0.7 mL) and ethanol (1 mL) and homogenized for 2 min. Hexane (2 mL) was added, and the mixture was further homogenized for 2 min. The hexane phase containing the carotenoids, Vitamins E and A, and coenzyme Q_10_ were separated by centrifugation, and the upper layer was collected. Extraction with 2 mL hexane was repeated twice, and the extracts were combined, evaporated, and re-dissolved in a mixture of methanol/dichloromethane (1:1 v/v). Samples were injected into a Shimatzu Prominence LC-20A Series-HPLC System (Istanbul, Turkey). Total carotenoids were identified by injecting 10-µl extracts into a Hypersil APS-2 5μ C18 reverse-phase HPLC column (25 cm × 4.6 mm, Phase Separations, Thermo Fisher Scientific, Waltham, MA, USA) with a mobile phase of methanol/water (97:3 v/v) at a flow rate of 1.5 mL/min. The HPLC was calibrated using standard lutein.

Vitamin E and A was identified using the same HPLC system with a Fluorescence Spectrofluorometer fitted with a Hypersil Gold 5μ C18 reverse-phase column (10 cm × 4.6 mm; Thermo, USA) and a mobile phase of methanol/water (97:3 v/v) at a flow rate of 1.05 mL/min and excitation and emission wavelengths of 295 and 330 nm. A standard solution of α-tocopherol and retinol in methanol were used for calibration. Coenzyme Q_10_ was analyzed by injecting 50 µL into the same HPLC system, but using a Vvdac 201TP54 column (5 µm, 25 cm × 4.6 mm, Radnor, PA, USA) and a mobile phase of ethanol/methanol/2-propanol (70:15:15 by volume), with a flow rate of 1.5 mL/min and PDA detection at 275 nm [36] and a standard coenzyme Q_10_ (Merck, Darmstadt, Germany) used for calibration. 

### 2.4. Statistical Analyses

**Figure 1:**
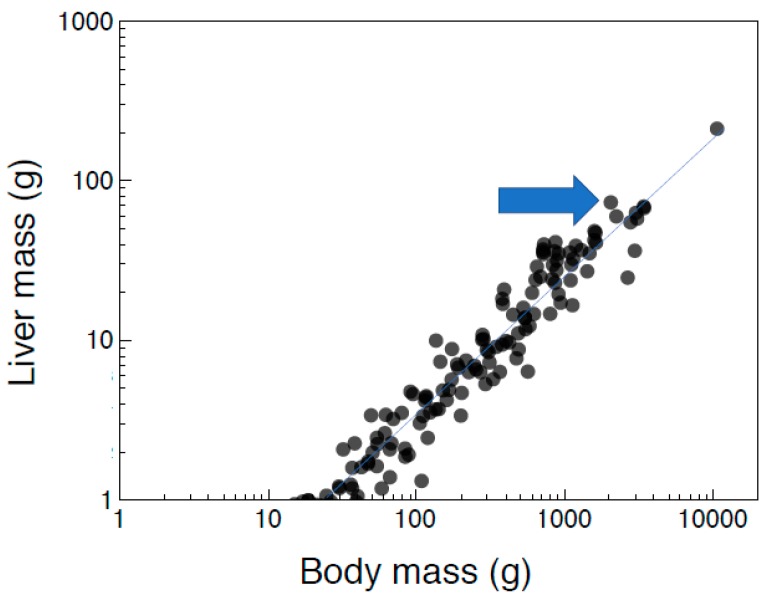
Liver mass (g) in 178 species of birds in relation to body mass (g) with the line being the allometric relationship and the observation at the blue arrow being the common eider *Somateria mollissima*. The line is the log-log regression line.

We estimated the interspecific allometric relationship between log_10_-transformed mean liver mass and log_10_-transformed body mass across 178 species of birds (Figure 1). We reported summary statistics (mean (SE)) for liver mass and antioxidants. We estimated the correlation matrix between liver mass, body mass, and antioxidants. We estimated correlations among variables to avoid problems of multicollinearity. However, variance inflation factors were all less than 3, implying that there were no problems of collinearity [37].

We developed Generalized Linear Models (GLM) including all the main predictors and all two-way interactions. We included sex and age in these models to assess whether they caused significant heterogeneity, and if that was not the case, these variables were subsequently deleted. This backward selection procedure was continued until all two-way interactions were eliminated and all remaining predictors had associated *p*-values < 0.05. 

Birds acquire their antioxidants through ingestion by the use of their beaks. Larger beaks ingest and process more food, and we should thus expect a positive association between the amount of antioxidants and beak volume. We tested these predictions using GLM with normally distributed data and an identity link function. Because large individuals generally have larger skeletal characters, and because concentrations of antioxidants are larger in larger individuals, we tested for a significant relationship between antioxidant levels and femur length as a measure of body size. All analyses were made using JMP [38]. 

## 3. Results

### 3.1. Interspecific Allometry of Liver Antioxidants

Liver mass increased allometrically with body mass across 178 species of birds (Figure 1; LR χ^2^ = 568.44, slope (SE) = 0.90, df = 1, 176, r^2^ = 0.96, *p* < 0.0001). This allometric relationship can be used to predict the expected liver size for a species of a given body mass. The allometry coefficient of 0.90 (0.01) was significantly less than 1 in a one-sample *t*-test (*t* = 7.071, df = 176, *p* < 0.0001). This implies that species with large body mass had disproportionately small livers. Residual liver mass for the common eider was +0.169, as shown by the positive residual indicated by the blue arrow in Figure 1, with 95% confidence intervals of +1.408 and +1.972. Thus, the common eider had a predicted liver mass that was on average 72.314 g, which was significantly larger than predicted from the allometric relationship for all species of birds. 

**Figure 2:**
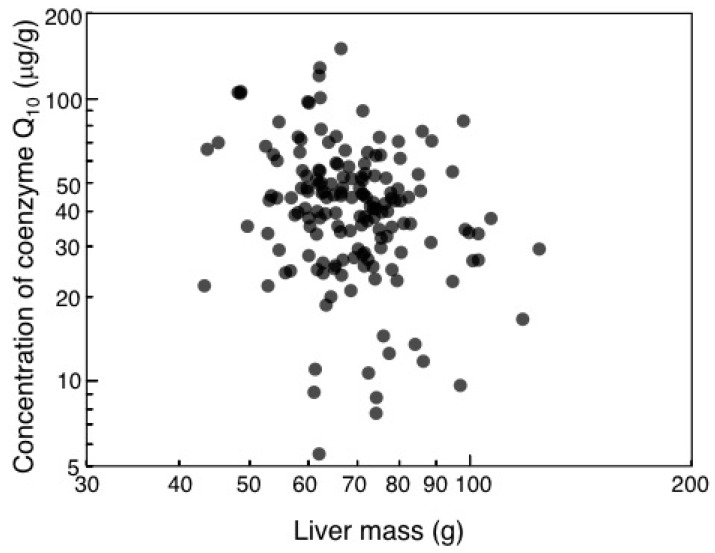
The concentration of coenzyme Q_10_ (μg/g) in the liver in relation to the mass of the liver in common eiders. The line is the log-log regression line.

Mean (SE) liver mass for the common eider was 70.14 g (1.05), *N* = 162, while body mass was 2.274 kg (0.014), *N* = 162. We tested whether mean antioxidant levels differed between the eider and the mean value for all other species of birds (Table 1). Concentrations of coenzyme Q_10_ (µg/g liver) decreased with increasing liver mass (Figure 2); likelihood ratio (LR) χ^2^ = 9.74, df = 1, *p* = 0.0018, estimate (SE) = −0.743 µg/g liver (0.235)), while liver mass was not significantly related to any other antioxidant. Total carotenoid concentration (µg/g liver) was significantly smaller in common eider (mean concentration = 4.295 µg/g liver) than in the mean value for the other species of birds studied here (44.134; *t* = −288.830, *p* < 0.0001). The concentration of Vitamin A in the liver did not differ significantly between the common eider (mean = 1.757 µg/g liver) and the mean for all other bird species (*t* = −1.928, *p* = 0.056). Vitamin E in liver was significantly smaller in common eider (mean = 2.900 µg/g liver) than for the mean of all other species of birds (mean = 120.327 µg/g liver; *t* = −599.78, *p* < 0.0001). 

### 3.2. Liver Antioxidants and Ecology of Common Eiders

The correlation matrix for liver mass, body mass, and antioxidant concentration showed few strong relationships. Because vitamin E can affect the absorption of vitamin A (and vice versa), this could affect the relationships. However, that was apparently not the case because eiders with large livers had low concentrations of coenzyme Q_10_ (Table 2). Individuals with high concentrations of vitamin A also had high concentrations of vitamin E, vitamin A was positively correlated with concentrations of carotenoids and coenzyme Q_10_, and total carotenoid concentration was positively correlated with coenzyme Q_10_ (Table 2). Thus, there was a preponderance of positive relationships between concentrations of different kinds of antioxidants. 

Animals acquire their antioxidants through ingestion by use of their beaks, and a larger beak can ingest and process a larger amount of food than a smaller beak [39,40,41,42]. Thus, we should expect a positive association between the amount of antioxidants and beak volume. Surprisingly, we only found a significant negative relationship, and that was only the case for three of four antioxidants. Concentrations of total carotenoids, vitamin E, and coenzyme Q_10_ decreased with beak volume (total carotenoids: LR χ^2^ = 8.81, df = 1, *p* = 0.0030, −13.258 µg/g liver (4.405); vitamin E: LR χ^2^ = 4.14, df = 1, *p* = 0.042, −0.0043 µg/g liver (0.0021); Q_10_: LR χ^2^ = 5.81, df = 1, *p* = 0.016, −0.0349 µg/g liver (0.144)). In contrast, there was no significant relationship between antioxidant levels and femur length, which is a standard skeletal measure of body size. 

The mass of the gizzard increased with increasing liver mass (LR χ^2^ = 7.95, df = 1, *p* = 0.0048, 0.000042 µg/g liver (0.00015)). The size of the gizzard decreased with the concentration of total carotenoids (LR χ^2^ = 8.09, df = 1, *p* = 0.0045, −0.0096 µg/g liver (0.0033)) and the concentration of coenzyme Q_10_ (LR χ^2^ = 6.86, df = 1, *p* = 0.0088, −0.117 µg/g liver (0.044)). 

### 3.3. Antioxidants, Date and Age

Antioxidant concentrations only differed between the sexes for total carotenoids with a higher value in males than in females (LR χ^2^ = 7.75, df = 1, *p* = 0.0054, 0.328 µg/g liver (0.012)). However, this effect of sex was not a confounding variable in the subsequent analyses because the effect of sex did not reach statistical significance in the full model. Body mass was the largest among individual eiders with high concentrations of total carotenoids (LR χ^2^ = 5.22, df = 1, *p* = 0.022, 0.019 µg/g liver (0.008)), while it was the lowest for concentrations of coenzyme Q_10_ with a low concentration in small individuals (LR χ^2^ = 4.05, df = 1, *p* = 0.0044, −0.0012 µg/g liver (0.0006)). Inclusion of femur length as an index of skeletal body size in the analysis of body mass did not change this conclusion. 

The concentration of total carotenoids in the liver increased with age (LR χ^2^ = 7.20, df = 1, *p* = 0.0073, 2.225 µg/g liver (0.820)), while the concentration of the other liver antioxidants was unrelated to age. The effect of sex was not significant and hence it was deleted from the final model. Vitamin E increased in relation to the October date (Figure 1a; LR χ^2^ = 10.97, df = 1, *p* = 0.0009, 0.026 µg/g liver (0.008)), as did total carotenoids (Figure 1b; LR χ^2^ = 33.06, df = 1, *p* < 0.0001, µg/g liver 0.027 (0.004)). However, there was no significant relationships with other antioxidants (vitamin A: LR χ^2^ = 2.99, df = 1, *p* = 0.08, slope (SE) = 0.003 µg/g liver (0.002)); Coenzyme Q_10_: LR χ^2^ = 1.14, df = 1, *p* = 0.286, 0.0008 µg/g liver (0.0008)). 

## 4. Discussion

The main findings of this study of antioxidants in the liver of a capital breeder, the common eider, were that liver size increased with body size and that the eider had a disproportionately large liver for its body size compared to other species. In contrast, the concentration of coenzyme Q_10_ decreased with liver size. Since coenzyme Q_10_ is the only endogenous fat-soluble antioxidant synthesized in the body from dietary intake, we can hypothesize that individuals with small livers for their body size are also those that have the smallest ability for such synthesis. 

Total liver mass increased allometrically with body mass with an allometry coefficient of 0.901 (SE = 0.014), and there was evidence that species with larger livers for their body size also have higher concentrations of antioxidants standardized to concentrations per gram of wet liver mass. Coenzyme Q_10_ has been hypothesized to be synthesized in response to oxidative stress, vitamin A deficiency, and the high level of physical activity associated with food acquisition. However, we found no explicit evidence of damage in this study. If the production of coenzyme Q_10_ is an adaptive mechanism against stressful conditions [43], this fits well with the scenario reported here that coenzyme Q_10_ concentration is highest among eiders with the smallest livers. This scenario also fits with the association between morphological traits involved in food ingestion and diet. 

Large eiders had high concentrations of total carotenoids in the liver, while there was a negative relationship between the concentration of coenzyme Q_10_ in the liver and body mass. These relationships were statistically independent of the October date and age, implying that total carotenoids either accumulated during winter, or that individuals with high levels of total carotenoids survived differentially. Similarly, the correlation between total carotenoids and age could either be due to individuals accumulating carotenoids with age or older individuals with higher concentrations of carotenoids surviving better. 

Eiders acquire their diet by use of their beaks, and individuals with large beaks should thus be able to acquire more food and hence more antioxidants [39,40,41,42]. The concentration of antioxidants (total carotenoids, vitamin E and coenzyme Q_10_) decreased among individuals with larger beaks. This may suggest that individuals with large beaks had difficulty acquiring sufficient antioxidants. Similarly, eiders with high concentrations of total carotenoids and coenzyme Q_10_ had small gizzards, suggesting that the processing of food by a large gizzard was associated with a low concentration of antioxidants. 

The main food items of eiders are blue mussels, draft whelks, and periwinkles. Previous research has shown that eiders with many blue mussels in the gizzard possess superior body condition [32]. Here we have shown that eiders that had consumed more draft whelks and beach crabs had larger livers. This had consequences for the concentration of antioxidants since the concentration of vitamin E increased with the number of razor clams and the number of draft whelks. The concentration of total carotenoids increased with the number of beach crabs in the gizzard. Thus, there was a link between beak morphology used for the capture and handling of food, the gizzard used for processing food, and possibly body condition. We are fully aware of the fact that this study did not provide information on the level of oxidative stress, and this important deficit, although common to almost all other field studies, requires future research. 

## 5. Conclusions

Here we have documented that the concentration of antioxidants in eiders is positively linked to the size of the liver, the size of the beak, and the empty gizzard, and subsequently to the composition of the diet. The results presented here suggest how the morphology of eiders is linked to diet, the concentration of antioxidants, and subsequently the seasonal composition of the diet and age-dependent concentrations of antioxidants. 

## Figures and Tables

**Table 1:** Comparison of mean (SE) liver mass and concentration of antioxidants in the livers of 162 common eiders. The table also contains information on the mean (SE) for 36 other species of birds for 2000–2016 for comparison with the common eider.

Variable	Mean for Eider	SE	*N*	Mean for All Other Species	SE	*N*
Liver mass (g)	70.117	1.065	162	12.494	1.617	179
Total carotenoids	4.295	0.142	162	44.134	5.408	36
vitamin A	1.539	0.113	162	1.751	0.202	36
vitamin E	2.900	0.201	162	120.327	12.500	36
Coenzyme Q_10_	45.160	1.883	162			

**Table 2:** Pearson product-moment correlation coefficients between liver mass, body mass, and concentration (µg/g) of antioxidants in livers of common eiders. Numbers in bold are statistically significant at the 0.05 level. The sample size was 162 eiders.

	Liver Mass					
Liver mass	1.000	Body mass				
Body mass	**0.270**	1.000	Vitamin A			
Vitamin A	0.085	0.088	1.000	Vitamin E		
Vitamin E	0.106	0.098	**0.658**	1.000	Total carotenoids	
Total carotenoids	−0.013	0.121	0.123	**0.525**	1.000	Coenzyme Q_10_
Coenzyme Q_10_	**−0.268**	−0.096	0.072	**0.291**	**0.391**	1.000
